# Influenza season 2012–2013 in Europe: moderate intensity, mixed (sub)types

**DOI:** 10.1017/S0950268814001228

**Published:** 2014-05-09

**Authors:** R. SNACKEN, E. BROBERG, J. BEAUTÉ, J. E. LOZANO, P. ZUCS, A. J. AMATO-GAUCI

**Affiliations:** 1 European Centre for Disease Prevention and Control (ECDC), Stockholm, Sweden; 2 Public Health Directorate General, Regional Ministry of Health. Government of Castilla y León, Valladolid, Spain

**Keywords:** Infectious disease epidemiology, influenza (seasonal), respiratory infections, surveillance system

## Abstract

This paper summarizes influenza activity in the European Union/European Economic Area (EU/EEA) in 2012–2013. The influenza season 2012–2013 in Europe lasted from early December to late April. Overall the severity of the season could be described as moderate, based on the ILI/ARI consultation rates and the percentage of sentinel specimens positive for influenza compared to previous seasons. Both influenza A and B viruses circulating accounted for 47% and 53% of positive sentinel specimens, respectively, with both A(H1) and A(H3) varying for dominance. Compared to outpatients, the proportion of laboratory-confirmed influenza hospitalized cases infected by A(H1N1)pdm09 was significantly higher in middle-aged patients (33% *vs*. 17%, *χ*^2^ = 66·6, *P* < 0·01). Despite a relatively good match between vaccine and circulating strains, vaccine effectiveness was estimated to be moderate.

Influenza is an acute respiratory disease of major public health importance that has been causing epidemics and pandemics of human disease for centuries, with significant morbidity and mortality still being reported every season [1]. A predominant feature of influenza is that its activity, severity and contagiousness vary in a most unpredictable manner each year as does the timing, intensity and duration of the annual epidemic wave. The objectives of the European Centre for Disease Prevention and Control's (ECDC) annual surveillance activities are to monitor the various elements of influenza virus activity (general epidemiological features and trends, intensity, geographical spread, circulating strains, antigenic and genetic characterization, antiviral susceptibility, severity in hospitalization, and mortality rates) in order to better guide public health prevention and control activities, vaccine strain selection, planning provision for patient care and eventually to reduce related morbidity and mortality.

Sentinel surveillance of influenza in European Union/European Economic Area (EU/EEA) countries is based on syndromic and virological data collected from primary- and secondary-care patients presenting with influenza-like illness (ILI) or acute respiratory infection (ARI). Twenty-seven EU member states, Iceland and Norway submitted their data weekly to ECDC from week 40 (2012) to week 20 (2013). The state of the season is estimated through qualitative reports by senior epidemiologists in scientific institutes or national influenza centres in the member states based on the intensity, geographical patterns and national trends of influenza activity. Nasopharyngeal swab specimens were taken from a systematic sample of sentinel ILI/ARI patients according to WHO [2] and national guidelines for sentinel sampling and were sent to reference influenza laboratories for detection and subtyping of influenza viruses. A subset of viruses were sent to specialized reference laboratories contracted by ECDC for further antigenic and genetic characterization, as well as antiviral susceptibility testing. In addition, eight countries reported data of hospitalized, laboratory-confirmed influenza cases. Descriptive analysis (means and proportions) and statistical inference (*χ*^2^ and Student's *t* tests) were performed using Stata v. 10.1 for Windows (StataCorp., USA) and R v. 3.0.1 with RStudio v. 0.97 (R Foundation, Austria).

Seasonal influenza surveillance started in the first week of October (week 40). In addition to higher intensity and/or wider geographical spread compared to baseline, a proportion of ⩾10% of specimens testing positive for influenza was reported by France, Ireland and the UK during week 48 (2012). Contrary to observations in previous years [3], the epidemic subsequently peaked in other European countries without any clear geographical progression pattern. The percentage of positive specimens peaked during weeks 4–12 (2013) when more than 50% of specimens were found positive for influenza (mean 57%, range 50–61%). It then dropped below 10% in week 17 (2013) (fourth week of April) for a total duration of 21 weeks. Overall, the percentage of influenza-positive specimens in 2012–2013 was substantially higher with the peak period lasting slightly longer than in previous non-pandemic seasons. In addition, in 18 countries, the ILI/ARI consultation rates in primary care were reported to be higher than in the 2011–2012 season, but similar to other previous seasons.

During the entire period under surveillance, the proportions of type A and B viruses varied over time and across Europe. For the overall season, of the 15 397 typed viruses from samples submitted through the sentinel system, 47% were type A and 53% type B. In 13 countries from central and northern Europe, influenza A viruses dominated throughout the season, i.e. accounted for >60% (range 61–94%) of the typed viruses, while B viruses dominated (range 64–76%) in Bulgaria, Italy, Ireland, Spain and the UK. The two viruses were co-dominant (both between 40% and 60%) in nine other countries (Fig. 1). In most instances, influenza B viruses circulated at the same time or later than the A viruses. In Ireland and the UK, circulation of influenza B viruses preceded circulation of A viruses, which is uncommon [4].

**Fig. 1.**
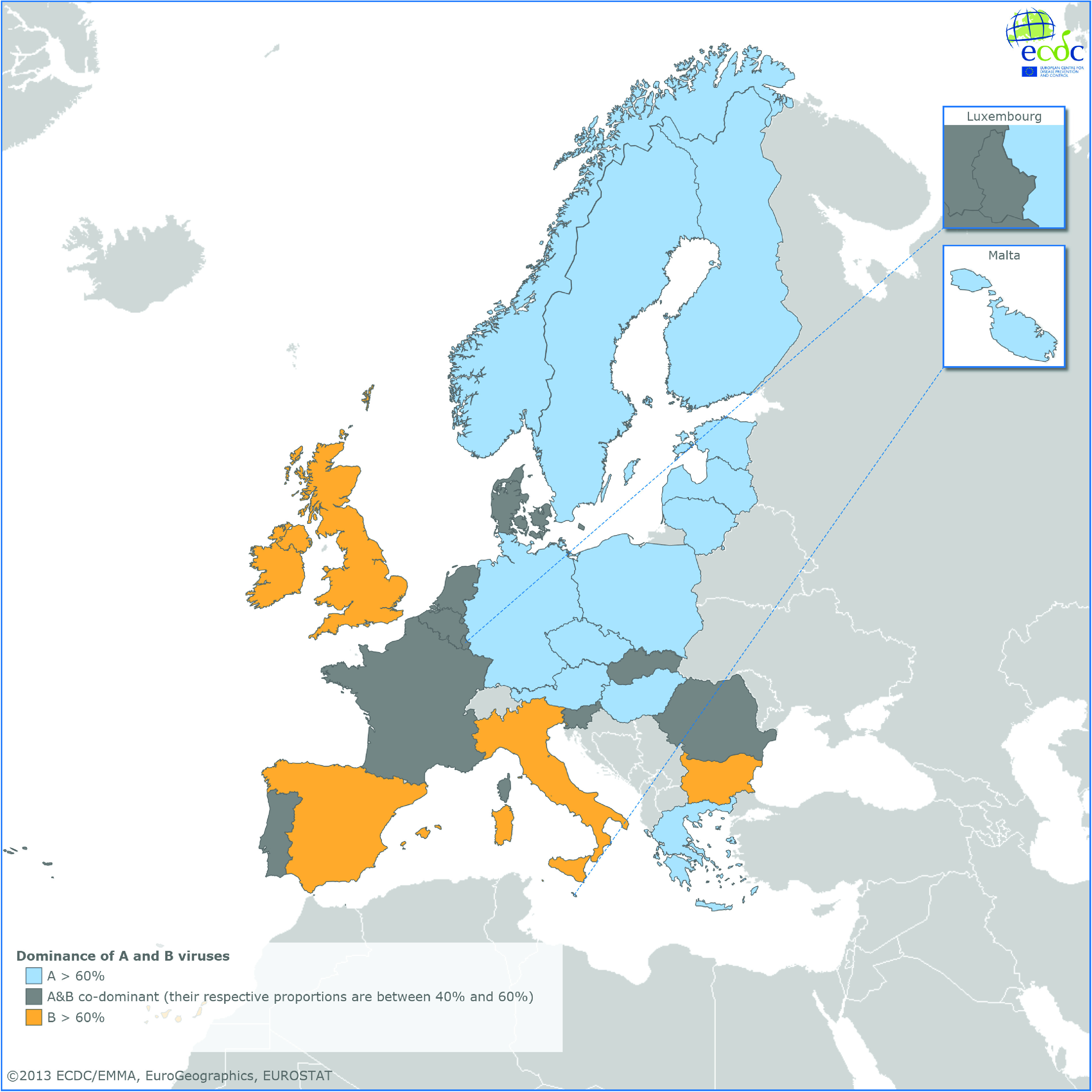
Dominance and co-dominance of A and B viruses from sentinel specimens, EU/EEA, 2012–2013.

Of the 6405 sentinel A viruses subtyped (a further 780 were classified as subtype unknown), 62% were A(H1N1)pdm09 but at country level, this proportion ranged from 16% in Denmark to 100% in Latvia. The proportion of A(H1N1)pdm09 was higher than 50% in 21 countries while A(H3N2) accounted for over 50% only in Denmark, Estonia, Greece, Ireland and the UK. Of the 2998 B viruses ascribed to lineage (5214 were classified as unknown lineage), 90% were Yamagata and 10% Victoria.

Most A(H3N2) and B (Yamagata) viruses from 23 EU countries were antigenically similar to prototype vaccine viruses for the influenza season 2012–2013. However, some A(H1N1)pdm09 [5] and A(H3N2) viruses [6] were found to have undergone some degree of drift which could partly explain the moderate vaccine effectiveness (51–56%) observed in North America [7,8].

A total of 1374 sentinel and non-sentinel viruses (975 influenza A, 399 influenza B) submitted by 10 countries were tested for antiviral susceptibility. Eleven (2%) of the 612 A(H1N1)pdm09 viruses tested carried the mutation associated with reduced inhibition by oseltamivir and one by zanamivir. In addition, one type B virus showed reduced inhibition by oseltamivir.

Eight countries reported 3388 hospitalized laboratory-confirmed influenza cases including 224 influenza-related deaths in six countries. The proportion of influenza A viruses was 61%, while in primary-care settings of the same eight reporting countries, this proportion was 38% (*χ*^2^ = 530·9, *P* < 0·01), suggesting a higher pathogenicity of influenza A viruses. The proportion of hospitalized cases infected by A(H1N1)pdm09 was significantly higher in patients aged < 65 years (33% *vs*. 17%, *χ*^2^ = 66·6, *P* < 0·01), highlighting the importance of influenza vaccination of middle-aged adults at risk of complications.

Finally, all-cause mortality data collected by 14 EU countries [9] has shown excess deaths in the elderly possibly explained by the pattern of influenza activity this season in Europe even if other factors such as the long and cold winter may also have contributed.

There are as yet no agreed criteria for assessing the severity of an epidemic season in the EU. The US CDC described their season as ‘moderately severe’, based on the higher percentage of outpatient visits for ILI, higher rates of hospitalization, and more reported deaths attributed to pneumonia and influenza compared to recent years [10]. The EU/EEA 2012–2013 season appeared to be similarly moderately severe in terms of reported intensity, prolonged duration of higher ILI/ARI case load, and the relatively longer-lasting higher proportions of influenza-positive samples. Characterization of influenza A viruses showed some genetic drift from vaccine strains, resulting in lower vaccine effectiveness than ideal. Very few viruses tested showed mutations associated with reduced inhibition to neuraminidase inhibitors. This season typified the unpredictability of influenza and underlined the importance of maintaining a robust influenza surveillance system.

## References

[1] Cox NJ, Subbarao K. Influenza. Lancet 1999; 354: 1277–1282. 10520648 10.1016/S0140-6736(99)01241-6

[2] WHO. WHO Regional Office for Europe guidance for sentinel influenza surveillance in humans, 2011 (http://www.euro.who.int/en/health-topics/communicable-diseases/influenza/publications/2009/who-regional-office-for-europe-guidance-for-sentinel-influenza-surveillance-in-humans). Accessed 10 March 2014.

[3] Paget J, Influenza activity in Europe during eight seasons (1999–2007): an evaluation of the indicators used to measure activity and an assessment of the timing, length and course of peak activity (spread) across Europe. BMC Infectious Diseases 2007; 7: 141. 18047685 10.1186/1471-2334-7-141 PMC2216029

[4] Meijer A, Epidemiological and virological assessment of influenza activity in Europe during the winter 2005–2006. Eurosurveillance 2007; 12: 217–224. 10.2807/esm.12.09.00733-en 17991413

[5] ERLI-NET. Influenza virus characterization-Surveillance report May 2013 (http://ecdc.europa.eu/en/publications/Publications/influenza-virus-characterisation-may-2013.pdf). Accessed 20 March 2014.

[6] Bragstad K, Low vaccine effectiveness against influenza A(H3N2) virus among elderly people in Denmark in 2012/13 – a rapid epidemiological and virological assessment. Eurosurveillance 2013; 18: pii=20397. 23410258

[7] Skowronski DM, Interim estimates of influenza vaccine effectiveness in 2012–2013 from Canada's sentinel surveillance network, January 2013. Eurosurveillance 2013; 18: pii=20394. 10.2807/ese.18.05.20394-en 23399422

[8] CDC. Interim adjusted estimates of seasonal influenza vaccine effectiveness – United States, February 2013. Morbidity and Mortality Weekly Report 2013; 62: 119–124. 23425960 PMC4604884

[9] Kanieff M, The potential for a concerted system for the rapid monitoring of excess mortality throughout Europe. Eurosurveillance 2010; 15: pii=19697. 10.2807/ese.15.43.19697-en 21087579

[10] CDC. Influenza activity – United States, 2012–13 season and composition of the 2013–2014 influenza vaccine. Morbidity and Mortality Weekly Report 2013; 62: 473–479. 23760189 PMC4604847

